# Emergency laparotomy preoperative risk assessment tool performance: A systematic review^☆^

**DOI:** 10.1016/j.sipas.2024.100264

**Published:** 2024-10-31

**Authors:** Joseph N. Hewitt, Thomas J. Milton, Jack Jeanes, Ishraq Murshed, Silas Nann, Susanne Wells, Aashray K. Gupta, Christopher D. Ovenden, Joshua G. Kovoor, Stephen Bacchi, Christopher Dobbins, Markus I. Trochsler

**Affiliations:** a The University of Adelaide, Discipline of Surgery, The Queen Elizabeth Hospital, South Australia, Australia; b College of Medicine and Public Health, Flinders University, South Australia, Australia; c Department of Surgery, Royal Adelaide Hospital, South Australia, Australia; d Department of Surgery, Gold Coast University Hospital, Queensland, Australia

**Keywords:** General surgery, Emergency laparotomy, Risk assessment

## Abstract

**Background:**

Preoperative assessment of risk for emergency laparotomy may enhance decision making with regards to urgency or perioperative critical care admission and promote a more informed consent process for patients. Accordingly, we aimed to assess the performance of risk assessment tools in predicting mortality after emergency laparotomy.

**Methods:**

PubMed, Embase, the Cochrane Library and CINAHL were searched to 12 February 2022 for observational studies reporting expected mortality based on a preoperative risk assessment and actual mortality after emergency laparotomy. Study screening, data extraction, and risk of bias using the Downs and Black checklist were performed in duplicate. Data on setting, operation undertaken, expected and actual mortality rates were extracted. Meta-analysis was planned but not possible due to heterogeneity. This study is registered with PROSPERO, CRD42022299227.

**Results:**

From 10,168 records, 82 observational studies were included. 17 risk assessment tools were described, the most common of which were P-POSSUM (42 studies), POSSUM (13 studies), NELA (12 studies) and MPI (11 studies). Articles were published between 1990 and 2022 with the most common country of origin being the UK (33 studies) followed by India (11 studies). Meta-analysis was not possible. Observed mortality and expected mortality based on risk assessment is reported for each study and generally shows most studies show accurate risk prediction.

**Conclusions:**

This review synthesises available literature to characterise the performance of various risk assessment tools in predicting mortality after emergency laparotomy. Findings from this study may benefit those undertaking emergency laparotomy and future research in risk prediction.

## Introduction

Emergency laparotomy, being any open abdominal procedure conducted on an unplanned or non-elective basis, encapsulates a broad range of procedures performed on a patient population extending in age from cradle to grave and in background from health to extreme illness. Contemporary mortality rate estimates at 30-days post operation for all comers span from 6.2 % [1] to 9.2 % [2]. From the heterogeneity in patients, pathology, operation and especially outcomes encapsulated by emergency laparotomy it follows logically that clinicians would be interested to predict an individual patient's outcome. Risk assessment or outcome prediction can play a key role in discussions with patients and their families, decisions on whether or not to operate and deciding access to limited resources such as theatre time or peri‑operative critical care admission. To this end multiple risk assessment tools have been developed. Use of such tools is advocated as best practice by the United Kingdom's National Emergency Laparotomy Audit (NELA) [2] and by the Royal Australasian College of Surgeons’ Australian and New Zealand Emergency Laparotomy Audit – Quality Improvement (ANZELA-QI) [1]. The use of preoperative risk assessment is one part of a large bundle of care which has seen 30-day mortality rates for emergency laparotomy fall from 12.7 % to 9.2 % over just eight years of the NELA [2]. The UK's NELA also includes laparoscopic procedures as laparotomies. One prospective study in the Australian setting found an association between risk assessment documentation and lower mortality rates [3] but this has not been borne out in other studies.

No available preoperative risk assessment tool has been shown to be clearly superior to another in emergency laparotomy. Accordingly, we performed a systematic review aiming to characterise the performance of preoperative risk assessment tools for emergency laparotomy in terms of accuracy in predicting mortality.

## Methods

We conducted a systematic review according to a protocol published *a priori* on PROSPERO (CRD42022299227) which included the research question, search strategy, inclusion and exclusion criteria and prespecified methods to assess methodological quality or risk of bias. Results are reported in accordance with the Preferred Reporting Items for Systematic Reviews and Meta-analyses 2020 (PRISMA 2020) reporting guidelines [4].

### Search strategy, inclusion and exclusion criteria

A research question was formulated in accordance with the PICO structure. The population for our review comprised patients undergoing emergency laparotomy. The intervention was the use of a preoperative risk assessment tool to predict mortality. The comparator was the use of other preoperative risk assessment tools. The primary outcomes were predicted and actual 30-day mortality. Only original observational studies were considered for inclusion. Case reports, editorials and literature reviews were excluded due to the low levels of evidence they present and the lack of original data.

PubMed, EMBASE, the Cochrane library and CINAHL were searched from database inception until 12th February 2022. PubMed was searched for (laparotomy) AND ((mort*) OR (death)) AND ((risk*) OR (scor*) OR (calculat*)). The Cochrane library was searched for laparotomy AND mortality AND risk. Embase was searched for (laparotomy) AND ((mortalty) OR (death)) AND ((risk) OR (score) OR (calculator)). CINAHL was searched for (Laparotomy) AND (mortality OR mortality rate OR death OR death rate) AND (risk OR score OR calculator). Results were restricted to human studies and those published in English. Results were supplemented with targeted searches of Google Scholar.

### Data extraction

Title and abstract screening were performed independently by two reviewers (JNH and JJ). Studies were uploaded to an online tool to facilitate the screening process (Rayyan, Qatar Computing Research Institute, Ar-Rayyan, Qatar). Two reviewers (JNH and IM) then independently screened full texts of studies. Disagreements at any stage of screening were resolved by a third reviewer (AG or SN). Data extraction was performed by two reviewers using a pre specified data extraction form (JNH and TJM) and disagreements resolved by consensus. Data were extracted for country, study design, operation and pathology characteristics, mortality, source of funding and reported conflicts of interest, methodological quality information, and other information relevant to the review questions.

### Data analysis

Data were synthesised in both narrative and tabular formats. Methodological quality was assessed using the Downs and Black risk of bias checklist [5] for non-randomised studies. Meta-analysis was planned but unfortunately was not possible due to significant heterogeneity between included studies.

## Results

**Fig. 1:**
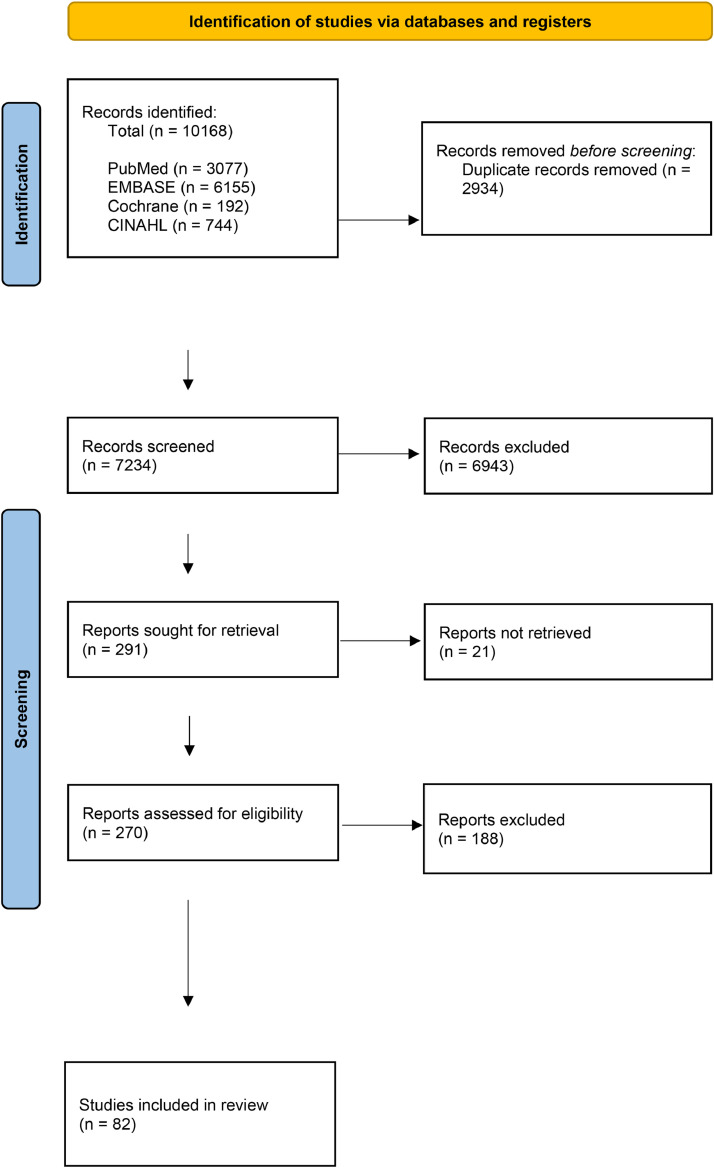
Study selection.

10,168 potentially relevant records were identified by the search strategy. 3077 were drawn from PubMed, 6155 from Embase, 192 from the Cochrane library and 744 from CINAHL. 82 studies were eventually included in the review. Targeted Google Scholar searches revealed no further studies. Fig. 1 shows a PRISMA flowchart of the study screening process.

### Settings and study design

**Fig. 2:**
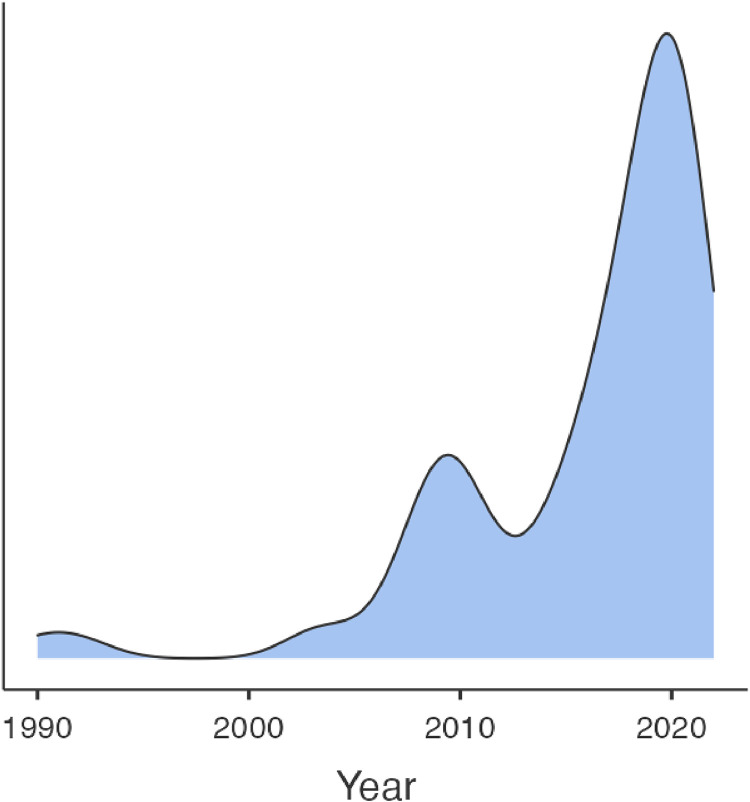
Frequency of study publication by year.

Articles were from between 1990 and 2022 with 2019 the median and 2020 the modal year of publication. Fig. 2 is a graphical representation of the frequency of year of publication of studies. Studies originated from 24 different countries. The most common country was the United Kingdom which contributed 33 studies. 11 were drawn from India, 9 from the USA, 4 from Australia, 3 from New Zealand and 3 from Italy. Remaining studies came from Canada, China, Finland, Germany, Greece, Indonesia, Ireland, Israel, Kenya, Malaysia, Pakistan, Singapore, Sweden, Switzerland, Tunisia and Turkey. 45 (55 %) studies were retrospective in nature. Sample size varied between 13 and 99,414 with a median of 196.

### Operations and outcome reporting

The majority of studies reported on emergency laparotomy of any type and indication. 4 studies were specific to laparotomy for perforated or bleeding peptic ulcer. 4 reported on laparotomy for diverticulitis only. 1 reported on laparotomy for large bowel obstruction, 1 for small bowel obstruction and 1 on laparotomy for any indication in patients on extra-corporeal membrane oxygenation (ECMO) support.

35 studies reported 30-day mortality. 25 studies reported mortality at hospital discharge. Remaining studies reported mortality at times varying between 28 days and 4 years.

### Risk assessment tools

The use of 17 preoperative risk assessment tools was reported. The most commonly used tool was P-POSSUM which was reported by 42 studies, followed by POSSUM and APACHE in 13 studies, NELA in 12 studies and MPI in 10 studies. Other tools used included ACS-NSQIP, Boey's score, BHOM, CELIOtomy, CR-POSSUM, ESS, IRCS, NFI, NS, POTTER, PULP, SORT and SPI. Included studies and characteristics are shown below in Table 1.

**Table 1:** Characteristics of included studies.

**Study (first author and year)**	**Country**	**Setting**	**Study design**	**Follow up**	**Operations or pathology included**	**n**	**Score(s) assessed**
Abbas [6]	New Zealand	Single hospital	Retrospective	30 days	Emergency laparotomy	1712	Simple Prognostic Index (SPI)
Aggarwal [7]	UK	Multicentre	Retrospective	30 days	Emergency laparotomy	13,953	P-POSSUM
Ah [8]	Sweden	Single hospital	Retrospective	30 days	Emergency laparotomy	209	P-POSSUM
Alder [9]	UK	Single hospital	Retrospective	19 months	Emergency laparotomy	153	P-POSSUM
Anbalakan [10]	Singapore	Single hospital	Retrospective	30 days	Perforated peptic ulcer	332	ASA, Boey's, Mannheim peritonitis index (MPI), Peptic ulcer perforation (PULP)
Barazanchi [11]	New Zealand	Single hospital	Retrospective	1 year	Emergency laparotomy	4419	NELA, P-POSSUM, ACS-NSQIP, APACHE-II
Barazanchi [12]	New Zealand	Single hospital	Retrospective	4 years	Emergency laparotomy	167	NELA
Basol [13]	Turkey	Single hospital	Retrospective	Hospital discharge	*Re*-laparotomies	236	APACHE-II
Body [14]	UK	Multicentre	Retrospective	1 year	Emergency laparotomy	610	NELA
Boyd-Carson [15]	UK and Wales	Multicentre	Retrospective	90 days	Emergency laparotomy	87,367	NELA
Boyd-Carson [16]	UK and Wales	Multicentre	Retrospective	30 days	Emergency laparotomy	33,819	P-POSSUM
Broughton [3]	Australia	Multicentre	Prospective	90 days	Emergency laparotomy	198	SORT
Burgess [17]	USA	Single hospital	Retrospective	Hospital discharge	Emergency laparotomy	95	ACS-NSQIP
Byrne [18]	UK and Wales	Multicentre	Retrospective	60 days	Bleeding/perforated peptic ulcer	2826	P-POSSUM
Cao [19]	Sweden	Single hospital	Retrospective	90 days	Emergency laparotomy	157	P-POSSUM
Chan [20]	Canada	Single hospital	Retrospective	Hospital discharge	Emergency laparotomy	211	APACHE-IV
Chatterjee [21]	India	Single hospital	Retrospective	Hospital discharge	Perforative peritonitis	50	POSSUM
Chieng [22]	Malaysia	Single hospital	Prospective	30 days	Emergency laparotomy	381	POSSUM, P-POSSUM
Choong [23]	Scotland	Single hospital	Prospective	1 year	Emergency laparotomy	462	NELA, P-POSSUM
Christou [24]	Greece and USA	Multicentre	Prospective	30 days	Emergency laparotomy	214	Emergency Surgery Score (ESS)
Clarke [25]	UK	Single hospital	Prospective	Hospital discharge	Emergency laparotomy	124	P-POSSUM
Coe [26]	UK and Wales	Multicentre	Retrospective	90 days	Perforated peptic ulcer	1158	NELA, P-POSSUM
Darbyshire [27]	UK and Wales	Multicentre	Retrospective	30 days	Emergency laparotomy	99,414	NELA, P-POSSUM
El-Hechi [28]	USA	Multicentre	Prospective	Hospital discharge	Emergency laparotomy	1347	ESS
El-Hechi [29]	USA	Multicentre	Retrospective	30 days	Emergency laparotomy	18,925	POTTER
El-Hechi 2020 (2) [30]	USA	Multicentre	Prospective	30 days	Emergency laparotomy	715	ESS
Eliezer [31]	Australia	Multicentre	Retrospective	30 days	Emergency laparotomy	562	NELA, P-POSSUM, ACS-NSQIP
Eugene [32]	UK and Wales	Multicentre	Retrospective	30 days	Emergency laparotomy	38,830	NELA, P-POSSUM, CR-POSSUM, SORT, IRCS, BHOM
Finlay [33]	UK	Single hospital	Prospective	Hospital discharge	Damage control surgery	14	POSSUM
Forse [34]	USA	Single hospital	Prospective	Hospital discharge	Laparotomy for intra-abdominal sepsis	13	APACHE-II
Garcea [35]	UK	Single hospital	Retrospective	Hospital discharge	Emergency laparotomy	280	P-POSSUM, APACHE-II
Hallam [36]	UK	Single hospital	Prospective	30 days	Emergency laparotomy	600	P-POSSUM
Haq [37]	Pakistan	Single hospital	Retrospective	8 weeks	Emergency laparotomy	150	POSSUM
Ho [38]	Australia	Single hospital	Retrospective	30 days	Emergency laparotomy	350	P-POSSUM
Horwood [39]	UK	Single hospital	Prospective	Hospital discharge	Emergency laparotomy and laparostomy formation	27	P-POSSUM
Huddart [40]	UK	Multicentre	Prospective	30 days	Emergency laparotomy	726	P-POSSUM
Jobin [41]	India	Single hospital	Prospective	28 days	Emergency laparotomy for perforation peritonitis	113	APACHE-II
Kaafarani [42]	USA	Multicentre	Prospective	30 days	Emergency laparotomy	1649	ESS
Kao [43]	USA	Single hospital	Retrospective	1 year	Emergency laparotomy	534	APACHE-II, MPI, CELIOtomy
Kimani [44]	Kenya	Single hospital	Prospective	30 days	Emergency laparotomy	166	POSSUM, P-POSSUM
Kumar [45]	India	Single hospital	Prospective	30 days	Emergency laparotomy	82	POSSUM, P-POSSUM
Lai [46]	Singapore	Multicentre	Prospective	30 days	Emergency laparotomy	830	NELA, P-POSSUM
Laitamaki [47]	Finland	Single hospital	Retrospective	1 year	Emergency laparotomy (palliative)	93	ACS-NSQIP
Leung [48]	UK	Single hospital	Prospective	30 days	Emergency laparotomy	619	P-POSSUM, CR-POSSUM
Livingstone [49]	UK	Single hospital	Retrospective	Hospital discharge	Emergency laparotomy	103	P-POSSUM
Malik [50]	India	Single hospital	Prospective	Hospital discharge	Emergency laparotomy	101	APACHE-II, MPI
McCann [51]	UK	Single hospital	Prospective	Hospital discharge	Emergency laparotomy on ECMO patients	13	P-POSSUM, APACHE-II
McIlveen [52]	UK	Single hospital	Prospective	1.5 years	Emergency laparotomy	214	P-POSSUM
Mohil [53]	India	Single hospital	Prospective	30 days	Emergency laparotomy	120	POSSUM, P-POSSUM
Mohil [54]	India	Single hospital	Prospective	Hospital discharge	Emergency laparotomy	101	P-POSSUM
Moore [55]	USA	Multicentre	Prospective	30 days	Emergent colon surgery	1147	APACHE-II
Mzoughi [56]	Tunisia	Single hospital	Retrospective	30 days	Emergency laparotomy	85	POSSUM
Nachiappan [57]	India	Single hospital	Prospective	Hospital discharge	Emergency laparotomy	97	POSSUM, MPI
Nag [58]	India	Single hospital	Prospective	30 days	Emergency laparotomy	157	P-POSSUM, APACHE-II
Nageswaran [59]	UK	Multicentre	Retrospective	60 days	Emergency laparotomy	1717	P-POSSUM
Neri [60]	Italy	Single hospital	Prospective	Hospital discharge	Perforative peritonitis	143	MPI
Nugent [61]	Ireland	Single hospital	Prospective	Hospital discharge	Emergency laparotomy	163	P-POSSUM
Parkin [62]	Australia	Single hospital	Retrospective	30 days	Emergency laparotomy	58	ACS-NSQIP
Parmar [63]	UK	Multicentre	Prospective	90 days	Emergency laparotomy	937	P-POSSUM
Pasternak [64]	Switzerland	Single hospital	Retrospective	Hospital discharge	Perforated diverticulitis	111	CR-POSSUM, MPI
Paul Trinity Stephen [65]	India	Single hospital	Prospective	Hospital discharge	Emergency laparotomy	78	MPI, APCHE-II
Peacock [66]	UK and Wales	Multicentre	Retrospective	Hospital discharge	Colonic perforation	6413	POSSUM
Peacock [67]	UK and Wales	Multicentre	Retrospective	30 days	Small bowel obstruction	9991	P-POSSUM
Peponis [68]	USA	Multicentre	Retrospective	30 days	Emergency laparotomy	26,410	ESS
Pinto-Lopes [69]	UK	Single hospital	Retrospective	30 days	Emergency laparotomy	43	P-POSSUM
Rivai [70]	Indonesia	Single hospital	Retrospective	Hospital discharge	Perforated peptic ulcer	72	Boey's
Salih [71]	UK and Wales	Multicentre	Retrospective	Hospital discharge	Emergency laparotomy	22,772	P-POSSUM
Saunders [72]	UK	Single hospital	Prospective	1 year	Emergency laparotomy	129	NELA, P-POSSUM
Schein [73]	German	Single hospital	Prospective	30 days	Emergency laparotomy	87	APACHE-II
Sharrock [74]	UK	Single hospital	Prospective	Hospital discharge	Emergency laparotomy	193	P-POSSUM
Singh-Ranger [75]	UK	Single hospital	Retrospective	30 days	Emergency laparotomy	91	P-POSSUM
Sohn [76]	Italy	Single hospital	Retrospective	Hospital discharge	Perforated diverticulitis	18	MPI
Spurling [77]	UK	Multicentre	Prospective	1 year	Emergency laparotomy	78,921	NELA
Sreeharsha [78]	India	Single hospital	Prospective	30 days	Emergency laparotomy	100	POSSUM
Stonelake [79]	UK	Single hospital	Retrospective	30 days	Emergency laparotomy	86	POSSUM, P-POSSUM, CR-POSSUM
Tartaglia [80]	Italy	Multicentre	Retrospective	Hospital discharge	Emergency laparotomy	34	MPI
Thahir [81]	UK	Single hospital	Retrospective	30 days	Emergency laparotomy	650	NELA, P-POSSUM
Trostchansky [82]	Israel	Single hospital	Retrospective	1 year	Emergency laparotomy	150	NS, MFI
Trotter [83]	UK	Multicentre	Prospective	1 year	Emergency laparotomy	259	P-POSSUM
Vashistha [84]	India	Single hospital	Retrospective	30 days	Emergency laparotomy	102	P-POSSUM
Zhang [85]	China	Single hospital	Retrospective	30 days	Bleeding	26	POSSUM, P-POSSUM
Zingg [86]	Switzerland	Single hospital	Retrospective	Hospital discharge	Perforated diverticulitis	111	CR-POSSUM, MPI

### POSSUM

Thirteen studies reported expected mortality based on APACHE scores. Year of publication ranged from 2003 to 2021. Twenty-nine originated from the UK, eight from India, two each from Australia, Sweden and Switzerland with other publications from individual countries. The expected and actual mortality for each study is shown in appendix A.

### APACHE

Thirteen studies reported expected mortality based on APACHE scores. Year of publication ranged from 1990 to 2021. Four studies originated from India, three from the USA, two from the UK with the remaining drawn from Canada, Germany, New Zealand and Turkey. The expected and actual mortality for each study is shown in appendix A.

### NELA

Twelve studies reported expected mortality based on NELA scores. Year of publication ranged from 2019 to 2022. Eight publications originated in the UK with two from New Zealand and one each from Australia and Singapore. The expected and actual mortality for each study is shown in appendix A.

### MPI

Ten studies reported expected mortality based on MPI scores. Year of publication ranged from 2008 to 2020. Three studies each were drawn from India and Italy with two from Switzerland and one each from the USA and Singapore. The expected and actual mortality for each study is shown in appendix A.

### ACS-NSQIP

Five studies reported expected mortality based on ACS-NSQIP scores. Year of publication ranged from 2017 to 2021. Two studies originated in Australia with one each from New Zealand, Finland and the USA. The expected and actual mortality for each study is shown below in appendix A.

### ESS

Five studies reported expected mortality based on ESS scores. Year of publication ranged from 2017 to 2021. Four were drawn from the USA and one included patients from the USA and Greece. The expected and actual mortality for each study is shown in appendix A.

### Other scoring systems

Scoring systems which were reported on by only one study have not been presented in tabular form but included Boey's score, BHOM, CELIOtomy, IRCS, NFI, NS, POTTER, PULP, SORT and SPI.

### Methodological quality

Methodological quality of included studies as assessed using the Down's and Black risk of bias tool is shown in Appendix A.

## Discussion

To our knowledge, this is the most up to date systematic review examining the performance of pre-operative risk assessment tools for emergency laparotomy. Accordingly, our findings may be of interest to clinicians undertaking emergency laparotomy or researchers examining pre-operative risk assessment. There is a large degree of heterogeneity present in our findings. Studies were published between 1990 and 2022. Although a modal year of publication of 2020 demonstrates an increasing interest in this topic, findings of older studies may be confounded by recent and ongoing advances not only in surgery but also in emergency medicine, anaesthesia and intensive care. 40 % of the studies originated from the United Kingdom but remaining studies are drawn from a range of different countries with vastly different healthcare systems. There is heterogeneity even in the definition of emergency laparotomy, noting that the UK's NELA includes laparoscopic procedures but these are excluded by ANZELA-QI. Reporting of post-operative mortality is variable based on what is considered the end-point of the post-operative period. Care should therefore be taken in extrapolating findings to different populations. Nevertheless, this review provides a summary of available literature.

The majority of studies in our review included patients undergoing emergency laparotomy of any type although a few focussed on specific populations, for example patients with perforated diverticular disease or patients on ECMO. This should be kept in mind if seeking to generalise results of these studies in particular. Large databases such as ANZELA-QI and NELA provide contemporary mortality rates for emergency laparotomy of 6.2 % [1] to 9.2 % [2], and in general the studies reviewed demonstrate comparable 30-day mortality. Where discrepancies arise there are potentially explained by time since publication and improvements in practice, practice setting, specific pathology investigated or study designs which focus on particular populations or on particularly high-risk subsets of patients.

Although meta-analysis was not possible as previously outlined, comparison of individual studies’ expected and observed mortality rates reveals generally accurate risk prediction by all tools assessed. As pooled comparison is not possible, a recommendation for use of a particular tool in preference to others cannot be advanced. This decision might also be influenced not just by accurate risk prediction but also by factors such as ease of use, availability of previous data, and factors specific to different localities and patient populations. Of particular relevance to the Australian and UK contexts is the accurate risk prediction demonstrated by the NELA tool which is recommended by ANZELA-QI and NELA audits. The lack of ability to undertake meta-analysis means surgeons may find it challenging to select the best risk assessment tool based on demonstrated superior accuracy. Consequently, the integration of these tools into practice should be approached with an understanding of their local applicability. Although P-POSSUM, NELA and APACHE were the most frequently utilised and have demonstrated accuracy, other factors including ease of use and local or departmental context may have greater influence over a clinician's choice of risk assessment tool. NELA is likely to be advanced as the risk assessment tool of choice in the Australian and UK contexts for example, due to the recommendation for its use by the nationally based ANZELA-QI and NELA audits. Large scale prospective collection of data from the same risk assessment tools in these contexts has obvious advantages in allowing greater comparison and promoting data homogeneity. The integration of preoperative risk assessment tools into existing clinical guidelines and protocols is a critical consideration for their use. Aligning these tools with established clinical pathways ensures a seamless incorporation into the broader framework of emergency laparotomy care. In the future, collaborative efforts between clinicians, policymakers, and researchers will be crucial in adapting these risk assessment tools and streamlining their use in local contexts to improve patient outcomes.

Our study has several limitations. There is extreme heterogeneity among studies in terms of location, operation, date of publication, and methods of reporting which whilst not unexpected, unfortunately made meta-analysis impossible. It is possible other studies which should have been included were not captured by our search strategy. Though quality of included studies was fair as assessed by the Downs and Black risk of bias tool, many included studies were retrospective in nature which increases the possibility of bias. The quality of evidence available on risk assessment performance could be enhanced by access to prospectively collected large scale data, such as that captured by NELA or ANZELA-QI databases.

Although emergency laparotomy is often a morbid procedure, it is very often also a lifesaving procedure. This study provides the most up to date synthesis of risk assessment accuracy for emergency laparotomy. Future research will benefit from further use of large scale prospectively collected databases such as ANZELA-QI and NELA and these will be crucial also in maintaining confidence in the accuracy of risk prediction tools as practice evolves, new tools are introduced, or existing tools improved. While accuracy is often a focus of the assessment of risk assessment tools, future research could also explore how the information derived from risk assessments influences patient experiences and satisfaction and if this differs between risk assessment tools. Additionally, integrating patient-reported outcomes into evaluation could offer a more holistic understanding of their impact on the overall emergency laparotomy experience.

## Source of funding

Joseph Hewitt is the recipient of an Australian Government Research Training Program scholarship.

## Data availability statement

Data supporting the study are available on request to the corresponding author, JNH.

## CRediT authorship contribution statement

**Joseph N. Hewitt:** Writing – review & editing, Writing – original draft, Project administration, Methodology, Investigation, Formal analysis, Data curation, Conceptualization. **Thomas J. Milton:** Writing – review & editing, Data curation. **Jack Jeanes:** Writing – review & editing, Data curation. **Ishraq Murshed:** Writing – review & editing, Data curation. **Silas Nann:** Writing – review & editing, Data curation. **Susanne Wells:** Writing – review & editing, Data curation. **Aashray K. Gupta:** Writing – review & editing, Data curation. **Christopher D. Ovenden:** Writing – review & editing, Data curation. **Joshua G. Kovoor:** Writing – review & editing, Data curation. **Stephen Bacchi:** Writing – review & editing, Data curation. **Christopher Dobbins:** Writing – review & editing, Supervision. **Markus I. Trochsler:** Writing – review & editing, Supervision.

## Declaration of competing interest

The authors declare the following financial interests/personal relationships which may be considered as potential competing interests: Joseph Hewitt reports financial support was provided by the Australian Government. Christopher Dobbins reports a relationship with Surgery in Practice and Science that includes: editorial board membership. The other authors declare that they have no known competing financial interests or personal relationships that could have appeared to influence the work reported in this paper.

## Appendix Supplementary materials

Image, application 1

## References

[1] Royal Australasian College of Surgeons (2022).

[2] NELA Project Team, Eighth Patient Report of the National Emergency Laparotomy Audit. 2023: London.

[3] Broughton K.J. (2017). The Perth Emergency Laparotomy Audit. ANZ J Surg.

[4] Page M.J. (2021). The PRISMA 2020 statement: an updated guideline for reporting systematic reviews. BMJ.

[5] Downs S.H., Black N. (1998). The feasibility of creating a checklist for the assessment of the methodological quality both of randomised and non-randomised studies of health care interventions. J Epidemiol Community Health.

[6] Abbas S.M. (2010). The Simple Prognostic Index (SPI)–a pathophysiologic prognostic scoring tool for emergency laparotomy. J Surg Res.

[7] Aggarwal G. (2020). Early postoperative death in patients undergoing emergency high-risk surgery: towards a better understanding of patients for whom surgery may not be beneficial. J Clin Med.

[8] Ah R. (2019). Prognostic value of P-POSSUM and osteopenia for predicting mortality after emergency laparotomy in geriatric patients. Bulletin of Emergency and Trauma.

[9] Alder L. (2021). Clinical frailty and its effect on the septuagenarian population after emergency laparotomy. Ann R Coll Surg Engl.

[10] Anbalakan K. (2015). Five year experience in management of perforated peptic ulcer and validation of common mortality risk prediction models - Are existing models sufficient? A retrospective cohort study. Int J Surg.

[11] Barazanchi A. (2020). Evaluating and improving current risk prediction tools in emergency laparotomy. Journal of Trauma & Acute Care Surgery.

[12] Barazanchi A. (2022). Short and long-term impact of sarcopenia on outcomes from emergency laparotomy. European journal of trauma and emergency surgery: official publication of the European Trauma Society..

[13] Basol O. (2016). Predictive factors affecting mortality in relaparotomies. Int J Clin Exp Med.

[14] Body S. (2021). Sarcopenia and myosteatosis predict adverse outcomes after emergency laparotomy: a multi-centre observational cohort study. Ann Surg.

[15] Boyd-Carson H. (2020). Trainee-led emergency laparotomy operating. Br J Surg.

[16] Boyd-Carson H. (2019). Association between surgeon special interest and mortality after emergency laparotomy. Br J Surg.

[17] Burgess J.R. (2017). Predicting postoperative complications for acute care surgery patients using the acs nsqip surgical risk calculator. Am Surg.

[18] Byrne B.E. (2018). Short-term outcomes after emergency surgery for complicated peptic ulcer disease from the UK National Emergency Laparotomy Audit: a cohort study. BMJ Open.

[19] Cao Y. (2020). The statistical importance of P-POSSUM scores for predicting mortality after emergency laparotomy in geriatric patients. BMC Med Inform Decis Mak.

[20] Chan T., Bleszynski M.S., Buczkowski A.K. (2016). Evaluation of APACHE-IV predictive scoring in surgical abdominal sepsis: a retrospective cohort study. Journal of Clinical and Diagnostic Research.

[21] Chatterjee A.S., Renganathan D.N. (2015). POSSUM: a scoring system for perforative peritonitis. Journal of Clinical and Diagnostic Research.

[22] Chieng T.H., Roslan A.C., Chuah J.A. (2010). Risk-adjusted analysis of patients undergoing laparotomy using POSSUM and P-POSSUM score in Queen Elizabeth Hospital, Sabah. Med J Malaysia.

[23] Choong J.X. (2021). Decision making in emergency laparotomy: the role of predicted life expectancy. BJS Open.

[24] Christou C.D. (2021). Validation of the Emergency Surgery Score (ESS) in a Greek patient population: a prospective bi-institutional cohort study. European journal of trauma and emergency surgery: official publication of the European Trauma Society..

[25] Clarke A. (2011). Mortality and postoperative care after emergency laparotomy. Eur J Anaesthesiol.

[26] Coe P.O. (2020). Open versus laparoscopic repair of perforated peptic ulcer disease: a propensity-matched study of the national emergency laparotomy audit. Ann Surg.

[27] Darbyshire A.R. (2022). P-POSSUM and the NELA score overpredict mortality for laparoscopic emergency bowel surgery: an analysis of the NELA database. World J Surg.

[28] El Hechi M. (2021). The Emergency Surgery Score accurately predicts the need for postdischarge respiratory and renal support after emergent laparotomies: a prospective EAST multicenter study. Journal of Trauma & Acute Care Surgery.

[29] El Hechi M.W. (2021). Validation of the Artificial Intelligence-Based Predictive Optimal Trees in Emergency Surgery Risk (POTTER) Calculator in Emergency General Surgery and Emergency Laparotomy Patients. J Am Coll Surg.

[30] El Hechi M. (2021). The emergency surgery score (ESS) and outcomes in elderly patients undergoing emergency laparotomy: a post-hoc analysis of an EAST multicenter study. Am J Surg.

[31] Eliezer D.D. (2020). High-Risk Emergency Laparotomy in Australia: comparing NELA, P-POSSUM, and ACS-NSQIP Calculators. J Surg Res.

[32] Eugene N. (2018). Development and internal validation of a novel risk adjustment model for adult patients undergoing emergency laparotomy surgery: the National Emergency Laparotomy Audit risk model. Br J Anaesth.

[33] Finlay I.G., Edwards T.J., Lambert A.W. (2004). Damage control laparotomy. Br J Surg.

[34] Forse R.A. (1992). Intra-abdominal sepsis and adrenergic receptor response. J Trauma.

[35] Garcea G. (2010). Preoperative early warning scores can predict in-hospital mortality and critical care admission following emergency surgery. J Surg Res.

[36] Hallam S. (2020). Does declared surgeon specialist interest influence the outcome of emergency laparotomy?. Ann R Coll Surg Engl.

[37] Haq M.Z., Ahmad N., Nasir I.I. (2012). Surgical audit of emergency surgery with the Possum system. Journal of Medical Sciences (Peshawar).

[38] Ho Y.M. (2018). Benchmarking against the National Emergency Laparotomy Audit recommendations. ANZ J Surg.

[39] Horwood J., Akbar F., Maw A. (2009). Initial experience of laparostomy with immediate vacuum therapy in patients with severe peritonitis. Ann R Coll Surg Engl.

[40] Huddart S. (2015). Use of a pathway quality improvement care bundle to reduce mortality after emergency laparotomy. Br J Surg.

[41] Jobin S.P. (2019). Role of serial lactate measurement to predict 28-day mortality in patients undergoing emergency laparotomy for perforation peritonitis: prospective observational study. J Intensive Care.

[42] Kaafarani H.M.A. (2020). Prospective validation of the Emergency Surgery Score in emergency general surgery: an Eastern Association for the Surgery of Trauma multicenter study. J Trauma Acute Care Surg.

[43] Kao A.M. (2020). The CELIOtomy Risk Score: an effort to minimize futile surgery with analysis of early postoperative mortality after emergency laparotomy. Surgery.

[44] Kimani M.M. (2010). Evaluation of POSSUM and P-POSSUM as predictors of mortality and morbidity in patients undergoing laparotomy at a referral hospital in Nairobi. Kenya. Annals of African Surgery.

[45] Kumar P., Rodrigues G.S. (2009). Comparison of POSSUM and P-POSSUM for risk-adjusted audit of patients undergoing emergency laparotomy. Ulus Travma Acil Cerrahi Derg.

[46] Lai C.P.T. (2021). A Comparison of the P-POSSUM and NELA Risk Score for Patients Undergoing Emergency Laparotomy in Singapore. World J Surg.

[47] Laitamäki M. (2021). Scoring Systems May be Effective in Predicting Mortality Associated with Palliative Emergency Gastrointestinal Surgery: a Retrospective Observational Study. World J Surg.

[48] Leung E. (2009). Predicting post-operative mortality in patients undergoing colorectal surgery using P-POSSUM and CR-POSSUM scores: a prospective study. Int J Colorectal Dis.

[49] Livingstone J. (2021). Comparing predicted and observed morbidity and mortality between emergency laparotomies conducted during the day and overnight at a district general hospital. European Surgery - Acta Chirurgica Austriaca.

[50] Malik A.A. (2010). Mannheim peritonitis index and APACHE II - Prediction of outcome in patients with peritonitis. Ulusal Travma ve Acil Cerrahi Dergisi.

[51] McCann C. (2019). Outcomes of emergency laparotomy in patients on extracorporeal membrane oxygenation for severe respiratory failure: a retrospective, observational cohort study. J Crit Care.

[52] McIlveen E.C. (2020). A prospective cohort study characterising patients declined emergency laparotomy: survival in the 'NoLap' population. Anaesthesia.

[53] Mohil R.S. (2004). POSSUM and P-POSSUM for risk-adjusted audit of patients undergoing emergency laparotomy. Br J Surg.

[54] Mohil R.S. (2008). Does nutritional status play a role in patients undergoing emergency laparotomy?. e-SPEN.

[55] Moore L.J. (2011). Availability of acute care surgeons improves outcomes in patients requiring emergent colon surgery. Am J Surg.

[56] Mzoughi Z. (2018). Abdominal emergency surgery in the elderly: how to predict mortality?. Journal of Clinical and Diagnostic Research.

[57] Nachiappan M., Litake M.M. (2016). Scoring systems for outcome prediction of patients with perforation peritonitis. Journal of Clinical and Diagnostic Research.

[58] Nag D.S. (2019). Comparative analysis of APACHE-II and P-POSSUM scoring systems in predicting postoperative mortality in patients undergoing emergency laparotomy. World J Clin Cases.

[59] Nageswaran H. (2019). Mortality for emergency laparotomy is not affected by the weekend effect: a multicentre study. Ann R Coll Surg Engl.

[60] Neri A. (2015). *Re*-evaluation of Mannheim prognostic index in perforative peritonitis: prognostic role of advanced age. A prospective cohort study. Int J Surg.

[61] Nugent E. (2021). Impact of service delivery factors on patient outcomes in emergency general surgery. surg.

[62] Parkin C.J. (2020). Utility of the American College of Surgeons National Surgical Quality Improvement Program surgical risk calculator in predicting mortality in an Australian acute surgical unit. ANZ J Surg.

[63] Parmar K.L. (2021). Frailty in Older Patients Undergoing Emergency Laparotomy: results From the UK Observational Emergency Laparotomy and Frailty (ELF) Study. Ann Surg.

[64] Pasternak I. (2010). Use of severity classification systems in the surgical decision-making process in emergency laparotomy for perforated diverticulitis. Int J Colorectal Dis.

[65] Paul Trinity Stephen D., Abraham V., Karuppusami R. (2020). Evaluation of Usefulness of Mannheim Peritonitis Index and APACHE II Score in Predicting Mortality and Morbidity in Patients with Peritonitis-A Prospective Diagnostic Test Study. Journal of Clinical and Diagnostic Research.

[66] Peacock O. (2021). Failure to rescue patients after emergency laparotomy for large bowel perforation: analysis of the National Emergency Laparotomy Audit (NELA). BJS Open.

[67] Peacock O. (2018). Thirty-day mortality in patients undergoing laparotomy for small bowel obstruction. Br J Surg.

[68] Peponis T. (2017). Does the emergency surgery score accurately predict outcomes in emergent laparotomies?. Surgery.

[69] Pinto-Lopes R., Thahir A., Halahakoon V.C. (2020). An Analysis of the Decision-Making Process After "Decision not to Operate" in Acutely Unwell, High-Risk General Surgery Patients. Am J Hosp Palliat Care.

[70] Rivai M.I., Suchitra A., Janer A. (2021). Evaluation of clinical factors and three scoring systems for predicting mortality in perforated peptic ulcer patients, a retrospective study. Annals of Medicine and Surgery.

[71] Salih T. (2021). Distance travelled to hospital for emergency laparotomy and the effect of travel time on mortality: cohort study. BMJ Quality and Safety.

[72] Saunders D. (2020). Postoperative Morbidity Survey: defined morbidity in emergency laparotomy patients. Anaesthesia.

[73] Schein M. (1990). Peritoneal lavage in abdominal sepsis. A controlled clinical study. Arch Surg.

[74] Sharrock A.E. (2017). Emergency abdominal surgery in the elderly: can we predict mortality?. World J Surg.

[75] Singh-Ranger D. (2017). Nontraumatic emergency laparotomy: surgical principles similar to trauma need to be adopted?. South Med J.

[76] Sohn M. (2016). Damage control strategy for the treatment of perforated diverticulitis with generalized peritonitis. Tech Coloproctol.

[77] Spurling L.J., Moonesinghe S.R., Oliver C.M. (2022). Validation of the days alive and out of hospital outcome measure after emergency laparotomy: a retrospective cohort study. Br J Anaesth.

[78] Sreeharsha H. (2014). Efficacy of POSSUM score in predicting the outcome in patients undergoing emergency laparotomy. Pol Przegl Chir.

[79] Stonelake S., Thomson P., Suggett N. (2015). Identification of the high risk emergency surgical patient: which risk prediction model should be used?. Annals of Medicine and Surgery.

[80] Tartaglia D. (2019). Damage control surgery for perforated diverticulitis with diffuse peritonitis: saves lives and reduces ostomy. World J Emerg Surg.

[81] Thahir A. (2021). Mortality risk scoring in emergency general surgery: are we using the best tool?. J Perioper Pract.

[82] Trostchansky I. (2019). Is Norton Score a useful tool for identifying high-risk patients prior to emergency surgery?. ANZ J Surg.

[83] Trotter J. (2018). Is sarcopenia a useful predictor of outcome in patients after emergency laparotomy? A study using the NELA database. Ann R Coll Surg Engl.

[84] Vashistha N. (2018). Outcomes of Emergency Laparotomy (EL) Care Protocol at Tertiary Care Center from Low-Middle-Income Country (LMIC). World J Surg.

[85] Zhang W.B. (2010). Risk factors of mortality in non-trauma exsanguinating patients that require damage control laparotomy. ANZ J Surg.

[86] Zingg U. (2010). Primary anastomosis vs Hartmann's procedure in patients undergoing emergency left colectomy for perforated diverticulitis. Colorectal Dis.

