# Supplementary material for: Emergency laparotomy preoperative risk assessment tool performance: A systematic review
Source: Surg Pract Sci. 2024 Oct 31;19:100264. doi: 10.1016/j.sipas.2024.100264 (PMC11750015; doi:10.1016/j.sipas.2024.100264)
Supplement: Supplementary file 1 [file mmc1.docx]

**Emergency laparotomy preoperative risk assessment tool performance: a systematic review**

**Authors:**

Joseph N. Hewitt^1,3^, MBBS, MMed; Thomas J. Milton^3^, MBBS, MS; Jack Jeanes^2^; Ishraq Murshed^1^, MBBS, MS; Silas Nann^4^, MBBS; Susanne Wells^1^; Aashray K. Gupta^4^, MBBS, MS; Christopher D. Ovenden^3^, MBBS, MS; Joshua G. Kovoor^1^, MBBS; Stephen Bacchi^1^, MBBS, PhD; Christopher Dobbins^3^, MBBS, MS, FRACS; Markus I. Trochsler^1^, MD, MMIS, FRACS

**Affiliations:**

1. The University of Adelaide, Discipline of Surgery, The Queen Elizabeth Hospital, South Australia, Australia
2. College of Medicine and Public Health, Flinders University, South Australia, Australia
3. Department of Surgery, Royal Adelaide Hospital, South Australia, Australia
4. Department of Surgery, Gold Coast University Hospital, Queensland, Australia

**Author for Correspondence:** Joseph N. Hewitt, Discipline of Surgery, The Queen Elizabeth Hospital, University of Adelaide, 28 Woodville Road, Woodville, SA 5011, Australia. Email: joseph.hewitt@adelaide.edu.au

**Supplementary Materials - Index**

| **Supplementary Appendixes** |  |
| --- | --- |
| Appendix A - tabulated results | *page 2 - 9* |

APPENDIX A

**Table 1: expected mortality based on POSSUM/P-POSSUM/CR-POSSUM vs actual mortality**

| **Study** | **n** | **Expected mortality** | **Mortality (discharge)** | **Mortality (30-day)** | **Mortality (90-day)** | **Mortality (other)** |
| --- | --- | --- | --- | --- | --- | --- |
| Aggarwal 2020 | 13953 | P-POSSUM: 54.2 (died within 3 days). P-POSSUM: 6.2 (died after 3 days) | 9.80% | 8.90% |  |  |
| Ah 2019 | 209 | P-POSSUM: 6.55 |  | 31.10% |  |  |
| Alder 2021 | 153 | P-POSSUM: 10.6 |  | 13% | 20% | 30.7% (1 year). 35.3% (19 months) |
| Barazanchi 2020 | 4419 | P-POSSUM: 13.4% |  | 7.90% |  | 15.1% (1 year) |
| Boyd-Carson 2019 | 33819 | P-POSSUM: Group 1: 17.07. Group 2: 20.91. Group 3: 22.47. Group 4: 23.35 |  | Group 1: 9.5%, group 2: 14.2%, group 3: 11.1%, group 4: 12.7% |  |  |
| Byrne 2018 | 2826 | P-POSSUM: <5% (n=984) 34.8%, n=453 (16%), n=519 (18.4%), n=375 (13.3%), n=495 (17.5%). |  |  |  | 12.6% (60 day). 0.2% (In theatre) |
| Cao 2020 | 157 | P-POSSUM: 5.7 |  |  | 29.30% |  |
| Chatterjee 2015 | 50 | POSSUM: 17.9% | 18% |  |  |  |
| Chieng 2010 | 381 | POSSUM: 18.656. P-POSSUM: 9.470 |  | 6.8% |  |  |
| Choong 2021 | 462 | P-POSSUM: <5%: n=200; >/=5%: n=261 |  | 11.30% | 16% | 14.1% (60 days). 23.2% (1 year) |
| Clarke 2010 | 124 | P-POSSUM: 27.4% | 19.10% | 16.90% |  |  |
| Coe 2020 | 1158 | P-POSSUM: 5.1% |  |  | 8.50% |  |
| Darbyshire 2022 | 99414 | P-POSSUM: 7.6% |  | 11.1% |  |  |
| Eliezer 2019 | 562 | P-POSSUM: 38.6% (non-survivors). 29.4% (survivors). |  | 10.5% |  |  |
| Eugene 2018 | 38830 | Presented graphically |  | 11.5% |  |  |
| Finlay 2003 | 14 | POSSUM: 23 | 7.10% | 0.0% |  |  |
| Garcea 2008 | 280 | POSSUM: 4.2 (survivors), 32.1 (non-survivors) | 15% |  |  |  |
| Hallam 2020 | 600 | P-POSSUM: Colorectal laparotomy by colorectal consultant: 4.5 (n=221), colorectal laparotomy by non-colorectal consultant: 10.3 (n=66). Oesophagogastric laparotomy by oesophogastric consultant: 12.45 (n=58), oesophgogatric laparotomy by non-oesophagogastric consultant: 6.6 (n=13). |  | 13.5% |  |  |
| Haq 2012 | 150 | POSSUM: 18% |  | 21.3% |  |  |
| Ho 2017 | 350 | P-POSSUM: 8.2% |  | 9.70% |  |  |
| Horwood 2009 | 27 | P-POSSUM: 72% (non-survivors). 48% (survivors) | 37% |  |  |  |
| Huddart 2014 | 726 | P-POSSUM: Group 1 (n=42) 19.7%. Group 2 (n=45) 22.3% |  | Group 1: 14%. Group 2: 10.5% |  |  |
| Kimani 2010 | 166 | POSSUM expected deaths: 28. P-POSSUM expected deaths: 12. |  | 4.80% | - |  |
| Kumar 2009 | 82 | POSSUM expected deaths: 17. P-POSSUM expected deaths: 11. |  | 9.8% |  |  |
| Lai 2021 | 830 | P-POSSUM: 16.3 |  | 5.7% |  |  |
| Leung 2009 | 619 | P-POSSUM: 11%. CR-POSSUM: 24% |  | 17% |  |  |
| Livingstone 2021 | 103 | P-POSSUM: Daylight hours: 5.6%, overnight: 2.7%. | Daylight hours 13.5%, overnight 9.3% |  |  |  |
| McCann 2021 | 13 | P-POSSUM: 91% | 62% | 62.0% |  |  |
| McIlveen 2019 | 214 | P-POSSUM: 17% |  | 13.0% |  |  |
| Mohil 2004 | 120 | POSSUM expected deaths: 26. P-POSSUM expected deaths: 24 |  | 13.0% |  |  |
| Mohil 2007 | 101 | P-POSSUM: 15.8% | 9% |  |  |  |
| Mzoughi 2018 | 85 | POSSUM: 30.7 (non-survivors), 21.2 (survivors) |  | 57.60% |  |  |
| Nachiappan 2016 | 97 | POSSUM: 40 (survivors), 58.5 (non-survivors). | 15.50% |  |  |  |
| Nag 2019 | 157 | P-POSSUM: 52 (non-survivors), <63 n=133 and >62 in n=1 (survivors) |  | 14.6% |  |  |
| Nageswaran 2019 | 1717 | P-POSSUM: Group 1: 6.5%. Group 2: 6% |  |  |  | 12.6% (60 days) |
| Nugent 2020 | 163 | P-POSSUM: 29.3 | 18% | 13% |  | 3.7% (7 day) |
| Parmar 2021 | 937 |  |  | Per P-POSSUM expected mortality. <5%, 5.6%; 5-10%, 12.6%; 10-20%, 12.9%; 20-50%, 23.1%; >/=50%, 38.1%. Overall 14.6% | 19.50% |  |
| Pasternak 2009 | 111 | CR-POSSUM: Hartman's: 27.2, primary anastomosis: 24.2. | Hartmann's (n=19) 29.2%, primary anastomosis (n=8) 17.4% |  |  |  |
| Peacock 2021 | 6413 |  | POSSUM <10: n=2638, mortality=2.3%; 10-24.9: n=1526, mortality 12.3%; 25-49.9: n=1106, mortality 25%; >/=50: n=1143, mortality=44.1%. Overall 16% |  |  |  |
| Peacock 2021 | 9991 |  |  | POSSUM <10, 2.7% mortality; 10-24.9, 12.7%; 25-49.9, 19.3%; >/=50, 35.2% |  |  |
| Pinto-Lopes 2019 | 43 | P-POSSUM: 12.3 |  | 18.6% |  |  |
| Salih 2020 | 22772 | P-POSSUM: Group 1: 21, Group 2: 21.37, Group 3: 22.46. |  | Overall 14.6%. Group 1: (n=1745) 14.1%. Group 2: (n=1186) 13.9%. Group 3: (n=293) 15.5%. | 17.90% |  |
| Saunders 2020 | 129 | P-POSSUM: 5.7% | 10.10% |  |  |  |
| Sharrock 2016 | 193 | P-POSSUM: 27.5 | 57.40% | 12.4% |  |  |
| Singh-Ranger 2017 | 91 | P-POSSUM: 5.6 (survivors), 82.8 (non-survivors) |  | 15.40% |  |  |
| Sreeharsha 2014 | 100 | POSSUM: 20% |  | 15.0% |  |  |
| Stonelake 2019 | 86 | POSSUM: 29.5%. P-POSSUM: 18.5%. CR-POSSUM: 10.5%. |  | 10.5% |  |  |
| Thahir 2020 | 650 | P-POSSUM: 15.2% |  | 9.1% |  |  |
| Trotter 2018 | 259 | P-POSSUM: 15% (sarcopenic), 7% (non-sarcopenic) |  | 13.90% |  | 28.2% (1 year) |
| Vashistha 2017 | 102 | P-POSSUM: 30.6% |  | 18.6% |  |  |
| Zhang 2008 | 26 | POSSUM: 77.7%, P-POSSUM: 63.4% |  | 26.9% |  |  |
| Zingg 2008 | 111 | CR-POSSUM: Hartmann's: 27, primary anastomosis: 24 | Hartmann's: 29.2%. Primary anastomosis: 17.4% |  |  |  |

**Table 2: expected mortality based on APACHE vs actual mortality**

| **Study** | **n** | **Expected mortality** | **Mortality (discharge)** | **Mortality (30-day)** | **Mortality (90-day)** | **Mortality (other)** |
| --- | --- | --- | --- | --- | --- | --- |
| Basol 2016 | 236 | APACHE-II: 26.06 | 15.3% |  |  |  |
| Forse 1992 | 13 | APACHE-II for survivors: 15.3. APACHE-II for non-survivors: 19 | 46.0% |  |  |  |
| Jobin 2019 | 113 | APACHE-II: 3 |  |  |  | 15% (28 days) |
| Moore 2011 | 1147 | APACHE-II: 31 |  | 28.3% |  |  |
| Schein 1990 | 87 | APACHE-II: 9 |  | 17.0% |  |  |
| Malik 2010 | 101 | APACHE-II: <10, 0% mortality, 10-20, 35.29%, >20, 91.7% | 16.8% |  |  |  |
| Kao 2020 | 534 | APACHE-II: 30 for patients who died before 72h, 16 for patients who survived beyond 72h | 13.9% |  |  | 27.5% (1 year) |
| Chan 2016 | 211 | APACHE-IV: 87.7 | 28.4% |  |  |  |
| Paul Trinity Stephen 2020 | 78 | APACHE-II: (non-survivors) 10.6 | 13.0% |  |  |  |
| Barazanchi 2020 | 4419 | 14.2% (APACHE-II) |  | 7.9% |  | 15.1% (1 year) |
| Garcea 2008 | 280 | APACHE-II: (survivors) 9. APACHE-II: (non-survivors) 12.5 | 15.0% |  |  |  |
| Nag 2019 | 157 | APACHE-II: (non-survivors) 31. APACHE-II: (survivors) 132 scored < 24 |  | 14.6% |  |  |
| McCann 2021 | 13 | APACHE-II: 21.7 | 62.0% | 62.0% |  |  |

**Table 3: expected mortality based on NELA vs actual mortality**

| **Study** | **n** | **Expected mortality** | **Mortality (discharge)** | **Mortality (30-day)** | **Mortality (90-day)** | **Mortality (other)** |
| --- | --- | --- | --- | --- | --- | --- |
| Barazanchi 2020[11] | 4419 | 7.4% |  | 7.9% |  | 15.1% (1 year) |
| Barazanchi 2021[12] | 167 | 10.9% |  | 13.2% |  | 24.6% (1 year), 43.1% (4 year) |
| Body 2022[14] | 610 | 4.5% |  | 7.7% |  | 18.9% (1 year) |
| Boyd-Carson 2020[15] | 87367 | 11.0% |  |  | 14.8% |  |
| Choong 2021[23] | 462 | 9.5% |  | 11.3% | 16.0% | 14.1% (60 days), 23.2% (1 year) |
| Coe 2020 | 1158 | 1.3% |  |  | 8.5% |  |
| Darbyshire 2022 | 99414 | 5.0% |  | 11.1% |  |  |
| Eliezer 2019 | 562 | 11.4% |  | 10.5% |  |  |
| Lai 2021 | 830 | 9.8% |  | 5.7% |  |  |
| Saunders 2020 | 129 | 3.7% | 10.1% |  |  |  |
| Spurling 2022 | 78921 | NELA predicted 30 day mortality groups. <5% 607/39502. 5-10% 933/12148. 10-20% 1685/10818. 20-30% 1438/5365. 30-40% 1137/3251. 40-50% 873/2006. >50% 1621/2753. |  | 11.0% | 15.1% | 18.3% (180 day), 22.5% (1 year) |
| Thahir 2020 | 650 | 7.8% |  | 9.1% |  |  |

**Table 4: expected mortality based on MPI vs actual mortality**

| **Study** | **n** | **Expected mortality** | **Mortality (discharge)** | **Mortality (30-day)** | **Mortality (90-day)** | **Mortality (other)** |
| --- | --- | --- | --- | --- | --- | --- |
| Malik 2010 | 101 |  | MPI: <15, 0%, 16-25, 4%, >25, 82.3% |  |  |  |
| Kao 2020 | 534 | MPI: (died within 72 hours) 27, (survived 72 hours) 19 | 13.90% |  |  | 27.5% (1 year) |
| Anbalakan 2014 | 332 | MPI: </=21, 3.1%, >22, 37.5% |  | 7.2% |  |  |
| Pasternak 2009 | 111 | MPI: Hartmann's (n=65), 21.2, primary anastomosis (n=46), 13.9 | Hartmann's: 29.2%, primary anastomosis: 17.4% |  |  |  |
| Zingg 2008 | 111 | MPI: Hartmann's (n=65), 21, primary anastomosis (n=46), 14 | Hartmann's: 29.2%, primary anastomosis: 17.4% |  |  |  |
| Neri 2014 | 143 | MPI <21: 5/68. MPI >/=21: 31/75. | 25.20% |  |  |  |
| Sohn 2016 | 18 | MPI: 17 | 11% |  |  |  |
| Tartaglia 2019 | 34 | MPI: 25.12 | 12% |  |  |  |
| Paul Trinity Stephen 2020 | 78 | MPI: (non-survivors) 32.5 | 13% |  |  |  |
| Nachiappan 2016 | 97 | MPI: (survivors) 24.5, (non-survivors) 33.5 | 15.50% |  |  |  |

**Table 5: expected mortality based on ACS-NSQIP vs actual mortality**

| **Study** | **n** | **Expected mortality** | **Mortality (discharge)** | **Mortality (30-day)** | **Mortality (90-day)** | **Mortality (other)** |
| --- | --- | --- | --- | --- | --- | --- |
| Burgess 2017 | 95 | 8.68% | 8.42% |  |  |  |
| Parkin 2020 | 58 | All patients >/=5% |  | 21.0% |  |  |
| Laitamaki 2021 | 93 | Median for patients who died 31.3%, median for patients who survived 18.3% |  | 41% | 63% | 22% (14 days). 87% (1 year) |
| Eliezer 2019 | 562 | 10.90% |  | 10.5% |  |  |
| Barazanchi 2020 | 4419 | 5.40% |  | 7.90% |  | 15.1% (1 year) |

**Table 6: expected mortality based on ESS vs actual mortality**

| **Study** | **n** | **Expected mortality** | **Mortality (30-day)** |
| --- | --- | --- | --- |
| Christou 2021 | 214 | ESS: (Greek population, n=102) 6, (US population, n=112) 8 | 16.0% |
| El-Hechi 2020 | 1347 | ESS: 5 | 0.7% |
| El-Hechi 2020 | 715 |  | ESS: 2, 0% mortality, 10, 20% mortality, 16, 60% mortality. Overall 21% |
| Kaafarani 2020 | 1649 | ESS: 6 | 14.80% |
| Peponis 2017 | 26410 |  | ESS: 1, 0.4% moratlity, 11, 39% mortality, 22, 100% mortality. Overall 10.2% |
